# Detection of Mycobacteria by Culture and DNA-Based Methods in Animal-Derived Food Products Purchased at Spanish Supermarkets

**DOI:** 10.3389/fmicb.2017.01030

**Published:** 2017-06-09

**Authors:** Iker A. Sevilla, Elena Molina, Maitane Tello, Natalia Elguezabal, Ramón A. Juste, Joseba M. Garrido

**Affiliations:** ^1^Animal Health Department, NEIKER-Instituto Vasco de Investigación y Desarrollo Agrario, Bizkaia Science and Technology Park 812LDerio, Spain; ^2^SERIDA-Servicio Regional de Investigación y Desarrollo Agrario, Carretera de OviedoVillaviciosa, Spain

**Keywords:** nontuberculous mycobacteria, *Mycobacterium tuberculosis* complex, dairy products, meat products, food contamination, prevalence

## Abstract

Mycobacteria include obligate and opportunistic pathogens that cause significant human and animal disease. The burden of tuberculosis has been largely reduced in developed territories but remains a huge problem worldwide. The significance of nontuberculous mycobacteria is growing considerably, especially in developed regions with higher life expectancy and more therapy-related immunosuppressed individuals. Due to their robustness mycobacteria can contaminate animal products by direct transmission from infected individuals or by environmental contamination during processing. The situation at market level is poorly known. Most studies analyzing commercially available foods are limited to a small or local scale and mainly focused on a particular mycobacterial species. There is a need to investigate if animal products that have passed the established controls to be for sale at main supermarkets could represent a route of contact with any mycobacteria. Thus, our goal was to study the prevalence of mycobacteria in these foods to assess if this could represent a source of human exposure. Five stores from the main supermarket chains in Spain were selected. 138 dairy and 119 meat products were purchased. All were processed using culture and multiplex real-time PCR methods. Additional molecular methods were used to specifically identify any positive result. *Mycobacterium avium* subsp. *hominissuis* (2), *M*. *avium* subsp. *avium* (1), and *M*. *fortuitum* (1) were isolated from powdered infant formula and ground beef, chicken sausage, and mortadella cold cut, respectively. Mycobacterial DNA (*M. avium, M. tuberculosis* complex and other nontuberculous mycobacteria) was detected in 15% of dairy products and 2% of meat products. These results show that the prevalence of viable mycobacteria in foods of animal origin obtained at the supermarket was not substantial although a considerable proportion of them contained mycobacterial DNA. Contact with mycobacteria through this route could be ensured over time. Further investigation is necessary to determine the real impact of foodborne mycobacterial exposure on human health and identify critical points in the food production system to enable setting up more stringent control measures.

## Introduction

The genus *Mycobacterium* includes important obligate and opportunistic infectious microorganisms that cause human and animal disease. *M. tuberculosis* complex together with *M*. *avium* complex and other nontuberculous mycobacteria (NTM) like *M*. *kansasii, M*. *malmoense, M*. *chelonae*-*abscessus* group, and *M*. *fortuitum* complex stand out among the rest of mycobacterial species in terms of medical and veterinary significance (Biet and Boschiroli, 2014; Falkinham, 2016). *M*. *tuberculosis* complex encompasses the etiological agents of human and zoonotic animal tuberculosis (TB), diseases of great health and socioeconomic impact as indicated in annual reports of the World Health Organization and manuals of the World Organization for Animal Health. *M*. *avium* subspecies are responsible for paratuberculosis (PTB), avian TB, infections in swine and other domestic and wild species, and cervical lymphadenitis, respiratory disease and disseminated or focal infections in humans (Biet and Boschiroli, 2014; Falkinham, 2016). *M*. *kansasii* is responsible for human disseminated infections and lung disease almost identical to typical TB (Griffith et al., 2007) and is commonly found in lymph node and tissue lesions in cattle in some regions (Biet and Boschiroli, 2014). The incidence of some ubiquitous fast growing NTM species (e.g., *M*. *fortuitum* complex and *M*. *chelonae*-*abscessus*) considered opportunistic pathogens for humans (Griffith et al., 2007) and animals (Bercovier and Vincent, 2001; Biet and Boschiroli, 2014) that originate diverse infections, has increased notably during the last decades (García-Martos and García-Agudo, 2012). In regions with developed health care resources the number of individuals susceptible to mycobacterial infection, especially with NTM, is growing due to the aging of the population, the maintenance of natural immune deficiencies and chronic diseases and the use of immunosuppressive therapies (Lake et al., 2016). Many mycobacterial infections originate from airborne exposure. However, other routes are also important, including the oral route.

Reports on foodborne (excluding water) mycobacterial transmission are mainly limited to zoonotic TB cases derived from the consumption of untreated or not properly treated *M*. *bovis*-contaminated foods. In developed territories infection through this route is not as frequent as in developing ones as a consequence of milk treatment and tuberculosis eradication programs (Pérez-Lago et al., 2014). But other mycobacterial infections can be contracted through oral exposure apart from TB. NTM cervical lymphadenitis affects mainly infants and there is circumstantial evidence indicating that infection originates from oral exposure (Kasperbauer and Huitt, 2013), most likely from water (Falkinham, 2003), and thus contaminated drinks and foods should not be discarded as possible vehicles. In most cases of NTM disseminated infection mycobacteria are introduced through the lungs or the digestive tract (Kasperbauer and Huitt, 2013). An association between NTM lung infection and gastroesophageal disorders has been previously recognized, being aspiration of contaminated water a probable means of bacterial introduction (Thomson et al., 2013). An etiologic role has been attributed to *M*. *avium* subsp. *paratuberculosis* in Crohn's disease but it remains controversial (Sechi and Dow, 2015). If this link is finally demonstrated, consumption of contaminated milk and dairy products could represent the most likely source of *M*. *avium* subsp. *paratuberculosis* (Grant, 2010). In ruminants the oral route is the main route of entry of this bacterium as is the case of *M*. *avium* subsp. *avium* in avian TB (Tell et al., 2001), and thus it could also represent a means of contact for humans.

The term environmental for NTM is determined by their presence in water, pipe systems, soil, dust and other ecosystems (Falkinham, 2016). Tuberculous mycobacteria and also NTM can contaminate foods of animal origin passing directly from infected individuals (including also their fluids and feces) to the product, but environmental contamination during the processing and storage is also possible (Grant, 2010; Ghodbane et al., 2014; Klanicova-Zalewska and Slana, 2014). In addition, as a result of their robustness mycobacteria can resist adverse conditions and survive to some of the disinfection or decontamination procedures applied to foods and drinks. In terms of microbiological safety and hygiene the presence of mycobacteria in foods represents a potential biological hazard that should be prevented. There is a lack of information with reference to supermarket foods that have passed the established controls. Many studies report on the detection of mycobacteria in animal tissues and raw milk and thus support that foods of animal origin may act as vehicles of mycobacteria transmission to humans (Kaneene et al., 2014; Klanicova-Zalewska and Slana, 2014; Pérez-Lago et al., 2014; Waddell et al., 2016). Several surveys have found mycobacterial DNA and viable cells in commercially available foodstuffs but the number of such reports is limited and most of them are mainly focused on the detection of *M*. *avium* subspecies or *M*. *bovis* (Grant, 2010; Klanicova et al., 2011; Faria et al., 2014; Lorencova et al., 2014; Pereira-Suarez et al., 2014; Savi et al., 2015; Cezar et al., 2016). According to the bibliography, animal products in which mycobacteria have been identified include raw and pasteurized milk, milk powder, powdered infant milk formula, cheese, meat, ground meat, fermented meat, ham, salami and sausages obtained from the main livestock species. Under these circumstances and taking into account the apparent increasing vulnerability of developed populations to some mycobacterial infections, we centered our attention on retail animal products available at the supermarket to determine whether they could represent a source of contact with mycobacteria for humans. For this purpose, processed and raw dairy and meat products purchased at different supermarkets were screened for the presence of members of the genus *Mycobacterium* using culture and PCR-based methods.

## Materials and methods

### Dairy and meat products

Five stores belonging to 5 of the main supermarket chains operating in Spain were selected. A total of 138 dairy and 119 meat products of different types and brands were collected at two sampling times. Samples covered top-selling brands along with own-label brands. Dairy foodstuffs included milk, milk powder, powdered infant formula, cream, butter, cheese and yogurt, and meat products consisted of packed ground meat, hamburger patties, fresh and cooked sausages, cold cuts, fresh Spanish chorizo and pâté. Most products were collected at both sampling times, thus two different batches of the same brand could be analyzed. All of them are frequently consumed products produced, processed, bottled or packed in Spain (89.9%), France (6.6%), Ireland (0.8%), Italy (0.4%), Germany (0.8%), Netherlands (0.8%), and Switzerland (0.8%). The number and type of products analyzed is summarized in Table 1. Brand names are not indicated to preserve the anonymity of the survey.

**Table 1:** List of food types and number of brands and items analyzed.

**Product**		**Obtained from (country)**	**Number of brands tested**			
			**First sampling**	**Second sampling**	**Both^a^**	**Total products**
Dairy products	Sterilized whole milk	Cattle (Spain)	1	1	1	2
	UHT whole milk	Cattle (Spain)	12	12	11	24
	Pasteurized whole milk	Cattle, sheep (Spain)	4	7	3	11
	Evaporated whole milk	Cattle (Spain)	0	1	0	1
	Milk powder (whole/skim)	Cattle (Spain)	4	4	4	8
	Powdered infant formula	Cattle (Spain, Netherlands, Switzerland)	5	5	5	10
	UHT milk cream	Cattle (Spain)	4	4	4	8
	Butter	Cattle (Spain, France, Germany)	7	7	7	14
	Pasteurized milk fresh cheese	Cattle (Spain, Italy)	12	13	12	25
	Pasteurized milk soft cheese	Cattle (Spain, France)	4	3	3	7
	Processed cheese	Cattle (Spain, France)	5	5	5	10
	Spreadable cheese	Cattle, goat (France)	2	2	2	4
	Pasteurized milk Greek style yogurt	Cattle (Spain, France)	7	7	7	14
	Total dairy products		67	71	64	138
Meat products	Ground meat	Cattle, pig, Iberian pig, chicken, turkey (Spain)	16	14	12	30
	Hamburger	Cattle, pig, Iberian pig, chicken, ostrich, horse, rabbit (Spain, Ireland)	9	13	7	22
	Fresh sausage	Pig, Iberian pig, chicken (Spain)	2	3	2	5
	Cooked sausage	Pig, Iberian pig, chicken (Spain)	3	4	3	7
	Mortadella cold cut	Pig (Spain)	3	3	3	6
	Headcheese cold cut	Iberian pig (Spain)	0	2	0	2
	Fresh Spanish chorizo	Pig, Iberian pig (Spain)	8	10	8	18
	Pâté	Pig; Iberian pig, wild boar, deer, duck, goose (Spain, France)	14	15	14	29
	Total meat products		55	64	49	119
All products	Total		122	135	113	257

*The country of production, processing, bottling or packing indicated on the product is included*.

a *Number of products tested in both sampling time points represented with two independent batches*.

### Culture

All samples were cultured in Lowenstein-Jensen with pyruvate (Difco, Francisco Soria Melguizo SA, Madrid, Spain), Coletsos (Difco), modified Middlebrook 7H10 (Becton, Dickinson and Company, MD, USA) with egg yolk and mycobactin J (IDvet, Grabels, France) prepared as previously described (Whittington et al., 2011) and in BBL Mycobacteria growth indicator tubes (MGIT) supplemented with BACTEC MGIT growth supplement and BBL MGIT PANTA (Becton, Dickinson and Company). Cultures on solid media were incubated at 37°C for 6 months and periodically inspected for the presence of colonies. MGIT cultures were incubated for 3 months in a BACTEC MGIT 960 System and time to detection (TTD) values recorded. Colonies on slants and MGIT cultures with a TTD value were submitted to PCR for verification and identification purposes. Different culture procedures were performed according to the sample type.

#### Milk, milk powder, and powdered infant formula

Powdered material was reconstituted with sterile distilled water following manufacturer's instructions and then treated like liquid milk. For solid cultures 30 ml of milk was centrifuged at 8,000 × g for 20 min. The pelleted material and the cream were homogenized and resuspended in 12 ml of 0.75% (wt/vol) RonaCare Cetylpyridinium Chloride (Merck, Darmstadt, Germany). Tubes were incubated on a rotating wheel at room temperature for 5 h (Donaghy et al., 2008). Samples were then centrifuged at 3,500 × g for 15 min. The intermediate liquid phase was discarded and the pellet and the remaining upper creamy fraction thoroughly vortexed with 1.5 ml of phosphate-buffered saline (PBS) containing 0.05% (wt/vol) Tween 20 (Sigma-Aldrich Co. Ltd., St. Louis, MO, USA). Solid media tubes were inoculated with 0.2 ml of this suspension. An additional 30 ml aliquot of samples was used for liquid culture. Following centrifugation at 8,000 × g for 20 min, the pellet and cream fractions were thoroughly homogenized in 10 ml of PBS-Tween 20. Afterwards, samples were processed with BD BBL MycoPrep kit (Becton, Dickinson and Company) following the instructions of the manufacturer and the resulting material was inoculated in appropriately supplemented MGIT tubes.

#### Cream, butter, cheese, and yogurt

Ninety ml of pre-warmed diluent containing 0.5% (wt/vol) sodium chloride, 2% (wt/vol) sodium citrate and 1% (wt/vol) Bacto Casitone (Difco, Becton, Dickinson and Company) were added to either 10 g of butter, 10 g of cheese or 10 g of yogurt contained in filter stomacher bags. For cream 30 ml of this product and 60 ml of diluent were used. Filter bags were introduced in a stomacher lab blender and suspensions homogenized for 3 min at high speed. After incubation at 56°C for 1 h on an orbital shaker platform, the homogenization step in the stomacher was repeated. Sixty ml of these suspensions were processed for solid (30 ml) and liquid (30 ml) culture following the steps previously described for milk samples. Ten milliliter of the same suspensions were used for DNA extraction.

#### Meat products

Filter stomacher bags were used to weigh 2 g of meat product. Following addition of freshly prepared RonaCare Cetylpyridinium Chloride (38 ml), samples were thoroughly homogenized in a lab blender and the suspension transferred to 50 ml centrifuge tubes. Tubes were then spun at 100 × g for 1 min and 15 ml of the supernatant containing no gross material was transferred to new centrifuge tubes. After an overnight decontamination, samples were centrifuged at 3,500 × g for 15 min and pellets resuspended in 1.5 ml of PBS-Tween 20. This was the suspension used to inoculate solid media tubes. An additional portion of 2 g was homogenized in a filter stomacher bag containing 10 ml of sterile distilled water. The homogenate was collected in 50 ml tubes and treated for MGIT culture as indicated before.

### DNA extraction

#### Dairy products

Adiapure milk kit (Adiagèn, bioMérieux, Marcy l'Etoile, France) was used as per manufacturer's instructions to extract DNA from milk and dairy product suspensions previously prepared for culture purposes (Donaghy et al., 2011). Briefly, a mixture consisting of 10 ml of sample and lysis buffer was vortexed and magnetic beads added. Tubes were gently mixed on a rotating wheel during 30 min for mycobacteria capture. Magnetic beads were separated from the liquid phase using a 15/50 ml tube magnet (Qiagen GmbH, Hilden, Germany), resuspended in the appropriate reagent and disrupted with glass beads for 10 min at maximum speed in a Tissue Lyser II (Qiagen) to lyse cells bound to magnetic beads. Beads were precipitated by centrifugation and supernatants treated with the buffer containing proteinase K.

#### Meat products

A modified protocol of the Speedtools Tissue DNA extraction kit (Biotools B&M Labs S. A., Madrid, Spain) was used (Sevilla et al., 2015). Meat product samples (2 g) were stomached with 10 ml of sterile distilled water in stomacher filter bags to give a homogeneous suspension. The material suspended in 1.25 ml was pelleted and the supernatant removed. Zirconia/silica beads of 0.1 mm diameter (Bio Spec Products Inc., Bartlesville, OK, USA), lysis buffer and proteinase K was added to tubes. Samples were incubated at 56°C with agitation in a ThermoMixer (Eppendorf, San Diego, CA, USA) until the suspension was completely clear. Incubation was extended with additional fresh proteinase K if this was not achieved at the first attempt. Subsequent processing was as the standard protocol of the kit indicated except for the inclusion of one bead-beating step in a Tissue Lyser II (Qiagen).

#### Positive cultures

One milliliter of MGIT broth was centrifuged at 16,000 × g for 3 min and the pellets washed with sterile distilled water. To extract DNA from these pellets and from the colonies grown on solid media the modified Speedtools Tissue DNA extraction kit (Biotools B&M Labs S. A.) protocol described above was employed.

All DNA extracts were stored at −20°C if not used immediately.

### Tetraplex real-time PCR screening

DNA from food products and cultures was tested using an improved version of a real-time PCR able to simultaneously detect the genus *Mycobacterium*, the *M*. *avium* subspecies, the *M*. *tuberculosis* complex and an internal amplification control (Sevilla et al., 2015). Modifications involved oligonucleotide changes in *M*. *avium* and *M*. *tuberculosis* complex PCR components to increase performance and accommodate the method to the use of different PCR mastermixes and platforms. The new protocol is currently under evaluation by an external party with commercial interests. Using the unmodified assay would yield similar results if performed under the same conditions reported earlier for the original method (Sevilla et al., 2015). Samples were tested in duplicate and all assays were validated based on the results obtained for internal amplification control, DNA extraction negative and positive controls, no template PCR controls and PCR positive controls. Amplification was carried out in a 7,500 Real-Time PCR instrument (Applied Biosystems, Foster City, CA, USA) under universal TaqMan assay conditions and SDS software v. 1.5.1 (Applied Biosystems) was used to calculate valid threshold cycle (C_T_) and baseline. C_T_ values can be used as a rough reference of the estimated numbers of mycobacteria that could be present in samples (Sevilla et al., 2015).

### Confirmation and further identification of samples and cultures positive to the tetraplex real-time PCR screening

#### Samples positive to *M. avium*

Samples positive for *M*. *avium* were submitted to IS*900*-IS*Map02* real-time PCR (Sevilla et al., 2014) and other conventional (Bartos et al., 2006) or real-time (Slana et al., 2010) PCR methods to detect IS*1245*, IS*901*, and IS*901*-flanking region (FR300). *M*. *avium* subspecies were identified on the basis of the presence (+) or absence (−) of the specified genomic targets as follows: *M*. *avium* subsp. *paratuberculosis* is IS*900*+, IS*Map02*+, IS*1245*−, IS*901*−, and FR300+; *M*. *avium* subsp. *avium* is IS*900*−, IS*Map02*−, IS*1245*+, IS*901*+ and FR300−; *M*. *avium* subsp. *silvaticum* is IS*900*−, IS*Map02*−, IS*1245*+, IS*901*+, and FR300+, *M*. *avium* subsp. *hominissuis* is IS*900*−, IS*Map02*−, IS*1245*+, IS*901*−, and FR300+ (Bartos et al., 2006).

#### Samples positive to *M. tuberculosis* complex

To confirm the positive results and distinguish between the species grouped in the *M*. *tuberculosis* complex, these DNA samples were analyzed by spoligotyping (Kamerbeek et al., 1997) and a panel of conventional singleplex PCR assays using primers and conditions previously described to detect the regions of difference (RD) 1, 4, 9, and 12 of *M*. *tuberculosis* (Halse et al., 2011). The RD signature patterns used to differentiate between *M*. *tuberculosis* complex members were those specified earlier (Halse et al., 2011). Briefly, *M*. *tuberculosis* is positive to all RDs; *M*. *canettii* is RD1+, RD4+, RD9+, and RD12−; *M*. *africanum* and *pinnipedii* are RD1+, RD4+, RD9−, and RD12+; *M*. *microti* is RD1−, RD4+, RD9−, and RD12+; *M*. *caprae* is RD1+, RD4+, RD9−, and RD12−; *M*. *bovis* is RD1+, RD4−, RD9−, and RD12−.

#### Samples positive to *Mycobacterium* sp. but negative to *M. avium* and *M. tuberculosis* complex

Species or complex identification and confirmation of samples only positive to the genus *Mycobacterium* was attempted by PCR and sequence analysis of 16S-23S rRNA internal transcribed spacer (ITS) (Richter et al., 1999) or by sequencing the short amplicon obtained for the ITS component (genus) in the screening PCR (Sevilla et al., 2015). Sequencing primers were the same used for amplification in both cases. Sequencing reactions were carried out using BigDye Terminator chemistry on a 3,130 Genetic Analyzer (Applied Biosystems). Sequences were inspected, edited and aligned with Sequencing Analysis 5.2 (Applied Biosystems) and Vector NTI (Informax Inc., Bethesda, MD, USA) software assistance and compared with other publicly available sequences using online BLAST (NCBI, NLM, Bethesda, MD, USA) analysis.

## Results

There was no agreement between PCR and culture positive results, no sample tested positive for both. Four dairy products that were positive in the direct PCR were also positive to the same PCR component when DNA extracted from MGIT culture was used, but viability could not be demonstrated as explained below.

### Culture results and identification of isolates

Colonies grown on solid media and MGIT cultures with positive TTD readouts on the BACTEC instrument were screened for the presence of mycobacterial DNA using the tetraplex PCR. All but six MGIT and two Middlebrook 7H10 cultures tested negative (see Table 2). However, four of these MGIT cultures yielded high C_T_ values (Table 2) and subculture attempts failed to demonstrate actual cell viability.

**Table 2:** Culture and screening tetraplex real-time PCR results of positive samples (detection of *M. avium, M. tuberculosis* complex and genus *Mycobacterium*).

**Product**	**From**	**Sample ID**	**Product culture (isolation medium)^a^**	**Product direct PCR (C_T_)^a^**	**Culture PCR (C_T_)^a^**
Pasteurized whole milk	Cattle	1	N	*Mycobacterium* (32.64)	ND
Whole milk powder	Cattle	2	N	*Mycobacterium* (43.62)	N
Pasteurized milk fresh cheese	Cattle	3	N	*Mycobacterium* (37.62)	N
Pasteurized milk fresh cheese	Cattle	4	N	*Mycobacterium* (36.42)	*Mycobacterium* (42.28)
Pasteurized milk fresh cheese	Cattle	5	N	*Mycobacterium* (38.31)	ND
Pasteurized milk fresh cheese	Cattle	6	N	*Mycobacterium* (38.98)	*Mycobacterium* (41.64)
Pasteurized milk soft cheese	Cattle	7	N	*Mycobacterium* (37.21)	ND
Pasteurized milk greek style yogurt	Cattle	8	N	*Mycobacterium* (38.46)	ND
Mortadella cold cut	Pig	9	P (MGIT)	N	*Mycobacterium* (25.6)
Pasteurized whole milk	Cattle	10	N	*M*. *avium* (39.1)	ND
Powdered infant formula	Cattle	11	N	*M*. *avium* (36.79)	*M*. *avium* (42.01)
Powdered infant formula	Cattle	12	P (M7H10)	N	*M*. *avium* (25.14)
UHT milk cream	Cattle	13	N	*M*. *avium* (38.77)	ND
Pasteurized milk fresh cheese	Cattle	14	N	*M*. *avium* (37.8)	ND
Pasteurized milk fresh cheese	Cattle	15	N	*M*. *avium* (36.74)	ND
Pasteurized milk fresh cheese	Cattle	16	N	*M*. *avium* (38.77)	N
Ground meat	Cattle	17	P (M7H10)	N	*M*. *avium* (14.34)
Hamburger	Horse	18	N	*M*. *avium* (37)	N
Fresh sausage	Chicken	19	N	*M*. *avium* (39)	N
Fresh sausage	Chicken	20	P (MGIT)	N	*M*. *avium* (14.90)
Whole milk powder	Cattle	21	N	*M*. *tuberculosis* complex (40)	N
Powdered infant formula	Cattle	22	N	*M*. *tuberculosis* complex (38.29)	N
Butter	Cattle	23	N	*M*. *tuberculosis* complex (40.03)	ND
Butter	Cattle	24	N	*M*. *tuberculosis* complex (38.08)	ND
Pasteurized milk fresh cheese	Cattle	25	N	*M*. *tuberculosis* complex (38.25)	N
Pasteurized milk soft cheese	Cattle	26	N	*M*. *tuberculosis* complex (40.12)	N
Pasteurized milk greek style yogurt	Cattle	27	N	*M*. *tuberculosis* complex (39.86)	*M*. *tuberculosis* complex (38.45)

a *P, positive; N, negative; ND, not done; C_T_, threshold cycle; M7H10, modified Middlebrook 7H10; MGIT, Mycobacterial Growth Indicator Tube*.

Identification of isolates is shown in Table 3. *M*. *avium* subsp. *hominisssuis* was isolated from a powdered infant formula (one colony on 7H10) as well as from one ground beef sample (one colony on 7H10). *M*. *avium* subsp. *avium* and *M*. *fortuitum* complex grew in MGIT cultures from a fresh chicken sausage and one mortadella sample, respectively. The ITS sequence obtained for the *M*. *fortuitum* complex isolate displayed a homology of 99% with *M*. *senegalense* strain MF-417 (82% query coverage) in BLAST analysis. No viable bacteria belonging to the *M*. *tuberculosis* complex were isolated.

**Table 3:** Confirmation and identification of samples positive to either culture or screening tetraplex real-time PCR.

**Product**	**From**	**Sample ID**	**Method used^a^**	**Results^a^^,^^b^**	**Identified as**
Pasteurized whole milk	Cattle	1	ITS sequencing^e^	*M*. *fortutitum* str.: 86–97%, 96–100%	*M*. *fortuitum* complex
Whole milk powder	Cattle	2	ITS sequencing^d^	*M*. *fortutitum* str.: 99%, 97%	*M*. *fortuitum* complex
Pasteurized milk fresh cheese	Cattle	3	ITS sequencing^d^	*M*. *terrae*/*M*. *arupense* str.: 100%, 100%	*M*. *terrae* complex
Pasteurized milk fresh cheese	Cattle	4	ITS sequencing^d^	*M*. *fortutitum* str.: 100%, 100%	*M*. *fortuitum* complex
Pasteurized milk fresh cheese	Cattle	5	ITS sequencing^d^	*M*. *gordonae* str.: 96%, 98%	*M*. *gordonae*
Pasteurized milk fresh cheese	Cattle	6	ITS sequencing^d^	*M*. *fortutitum* str.: 99%, 100%	*M*. *fortuitum* complex
Pasteurized milk soft cheese	Cattle	7	ITS sequencing^d^	*M*. *fortutitum* str.: 100%, 100%	*M*. *fortuitum* complex
Pasteurized milk greek style yogurt	Cattle	8	ITS sequencing^d^	*M*. *terrae*/*M*. *arupense* str.: 100%, 100%	*M*. *terrae* complex
Mortadella cold cut	Pig	9^c^	ITS sequencing^e^	*M*. *senegalense* str.: 99%, 82%	*M*. *fortuitum* complex
Pasteurized whole milk	Cattle	10	IS screening	IS*900*+, IS*Map02*+, IS*1245*−, IS*901*−, FR300+	*M*. *avium* subsp. *paratuberculosis*
Powdered infant formula	Cattle	11	IS screening	IS*900*+, IS*Map02*+, IS*1245*−, IS*901*−, FR300+	*M*. *avium* subsp. *paratuberculosis*
Powdered infant formula	Cattle	12^c^	IS screening	IS*900*−, IS*Map02*−, IS*1245*+, IS*901*−, FR300+	*M*. *avium* subsp. *hominissuis*
UHT milk cream	Cattle	13	IS screening	IS*900*+, IS*Map02*+, IS*1245*−, IS*901*−, FR300+	*M*. *avium* subsp. *paratuberculosis*
Pasteurized milk fresh cheese	Cattle	14	IS screening	IS*900*+, IS*Map02*+, IS*1245*−, IS*901*−, FR300+	*M*. *avium* subsp. *paratuberculosis*
Pasteurized milk fresh cheese	Cattle	15	IS screening	IS*900*+, IS*Map02*+, IS*1245*−, IS*901*−, FR300+	*M*. *avium* subsp. *paratuberculosis*
Pasteurized milk fresh cheese	Cattle	16	IS screening	IS*900*+, IS*Map02*+, IS*1245*−, IS*901*−, FR300+	*M*. *avium* subsp. *paratuberculosis*
Ground meat	Cattle	17^c^	IS screening	IS*900*−, IS*Map02*−, IS*1245*+, IS*901*−, FR300+	*M*. *avium* subsp. *hominissuis*
Hamburger	Horse	18	IS screening	IS*900*−, IS*Map02*−, IS*1245*+, IS*901*−, FR300+	*M*. *avium* subsp. *hominissuis*
Fresh sausage	Chicken	19	IS screening	IS*900*−, IS*Map02*−, IS*1245*+, IS*901*−, FR300+	*M*. *avium* subsp. *hominissuis*
Fresh sausage	Chicken	20^c^	IS screening	IS*900*−, IS*Map02*−, IS*1245*+, IS*901*−, FR300−	*M*. *avium* subsp. *avium*
Whole milk powder	Cattle	21	RD screening	RD1+, RD4−, RD9−, RD12−	*M*. *bovis*
Powdered infant formula	Cattle	22	RD screening	RD1+, RD4−, RD9−, RD12−	*M*. *bovis*
Butter	Cattle	23	RD screening	RD1+, RD4−, RD9−, RD12−	*M*. *bovis*
Butter	Cattle	24	RD screening	RD1+, RD4−, RD9−, RD12−	*M*. *bovis*
Pasteurized milk fresh cheese	Cattle	25	RD screening	RD1+, RD4−, RD9−, RD12−	*M*. *bovis*
Pasteurized milk soft cheese	Cattle	26	RD screening	RD1+, RD4−, RD9−, RD12−	*M*. *bovis*
Pasteurized milk greek style yogurt	Cattle	27	RD screening	RD1+, RD4−, RD9−, RD12−	*M*. *bovis*

a *+, positive; −, negative; ITS, 16S-23S rRNA internal transcribed spacer; IS, insertion sequences of M. avium; RD, regions of difference of M. tuberculosis complex; Str., strain*.

b *BLAST results displaying highest identity and coverage percentages for sequenced ITS amplicons: identity %, coverage %*.

c *DNA extracted from the isolate. Otherwise, the DNA was obtained directly from the product*.

d *Sequenced ITS amplicon corresponded to the tetraplex real-time PCR (Sevilla et al., 2015)*.

e *Sequenced ITS amplicon corresponding to the PCR described by Richter et al. (1999)*.

### Direct PCR detection in products and identification of positive samples

Mycobacterial DNA was detected in a total of 23 samples that accounted for the 15.22% of dairy products and 1.68% of meat products analyzed (Table 2). According to the tetraplex PCR results, 34.78% of positives corresponded to *M*. *avium*, 30.43% to *M*. *tuberculosis* complex and the remaining 34.78% to mycobacteria other than *M*. *avium* and *M*. *tuberculosis* complex. In meat foods only *M*. *avium* was detected. All but one product PCR positive samples displayed C_T_ values above 36 indicating the presence of very low amounts of detectable mycobacterial DNA. Regardless of these high C_T_ values, all positive results could be confirmed by the repetition of the screening PCR as well as by PCR testing of *M*. *avium* insertion sequences or *M*. *tuberculosis* complex RDs and mycobacterial ITS amplicon sequencing (see Table 3).

In this survey *M*. *tuberculosis* complex DNA was identified only in dairy products, including whole milk powder, infant formula, butter, cheese and yogurt. Samples recorded as *M*. *tuberculosis* complex positive were inspected for the presence of RD 1, 4, 9, and 12, and only RD1 was detected in all cases. This signature was suggestive of the presence of *M*. *bovis*. Spoligotyping failed to confirm this due to the absence of any perceptible hybridization signal.

DNA from *M*. *avium* subsp. *paratuberculosis* and *M*. *avium* subsp. *hominissuis* was identified in 6 dairy foods (pasteurized whole milk, infant formula, cream and fresh cheese) and 2 meat products (hamburger and sausage), as assessed by the presence or absence of the insertion sequences investigated. Samples deemed *Mycobacterium* sp. positive in the tetraplex PCR were finally classified as mycobacteria compatible with *M*. *fortuitum* complex (5), *M*. *terrae* complex (2) and *M*. *gordonae* (1) according to the sequences obtained.

With regard to foods represented by two product batches, only two displayed a positive result for both. However, the mycobacteria identified differed between batches. One powdered infant formula resulted positive to *M*. *tuberculosis* complex (*M*. *bovis*) in the first sampling and positive to *M*. *avium* (subsp. *paratuberculosis*) in the second. The other product was a fresh chicken sausage with batches positive to *M*. *avium* subsp. *hominissuis* DNA in one case and to *M*. *avium* subsp. *avium* culture in the other.

## Discussion

Direct PCR revealed the presence of mycobacteria in 9% of products, a proportion increased in dairy foodstuffs (15%) if compared with meat products (2%). All samples displayed very high C_T_ values close to or above those obtained at the limit of detection of the unmodified technique with artificially inoculated samples (100 CFU per gram) (Sevilla et al., 2015). The different nature of samples as well as the use of the modified method might account for a slightly different association between C_T_ values and limit of detection. In spite of this, the high C_T_ values were not PCR artifacts as was demonstrated by the confirmatory tests performed and therefore, low bacterial concentrations were to be expected in samples. The lack of agreement observed between PCR and culture can be explained by the heterogeneous distribution and low concentration of viable mycobacteria (probably below the limit of detection of PCR) in the products and the fact that DNA detection does not necessarily imply viability. Other factors that could account for PCR negative but culture positive results include a lower sensitivity of the DNA extraction and detection method or the presence of cell clumps.

*M*. *avium* subsp. *paratuberculosis* DNA was present in 4% of dairy foods. This figure is consistent with the lowest percentages of positives reported elsewhere (Grant, 2010; Waddell et al., 2016). In spite of this, we found less *M*. *avium* subsp. *paratuberculosis* than we anticipated based on the high prevalence and spread of PTB. In comparison to other reports on the culture of viable *M*. *avium* subsp. *paratuberculosis* from raw and pasteurized milk products (Waddell et al., 2016), culture did not produce any isolates. Our survey did not include any raw milk products and the number of pasteurized liquid milk samples was reduced. The sensitivity of DNA extraction and culture could represent an additional limitation that may be improved using novel methods (Botsaris et al., 2016). Despite this, a colony of *M*. *avium* subsp. *hominissuis* was isolated from a powdered infant formula. Little is known about the prevalence of this subspecies in dairy products. Among other infections, it is responsible for cervical lymphadenitis in infants and the most likely route of infection is oral (Falkinham, 2003; Kasperbauer and Huitt, 2013). Water is considered the main source of human exposure to this microbe (Falkinham, 2015) but infant formula could also represent a way of contact for children.

On the other hand, the detection of *M*. *tuberculosis* complex DNA in 5% of dairy products was completely unexpected. All cases were identified as *M*. *bovis* by RD screening. Spoligotyping was negative presumably because very small amounts and proportion of target DNA was present in DNA specimens obtained from food products. DNA extracted from these complex matrices can harbor a high concentration of nontarget DNA and other elements that may interfere with spoligotyping. The performance of spoligotyping can be limited also when applied directly to clinical samples (Milián Suazo et al., 2010; Ereqat et al., 2013). Our results did not seem to reflect the current PTB and TB situation in developed countries with high PTB prevalence and ongoing TB eradication and surveillance programs (Lombard, 2011; Schiller et al., 2011). It can be speculated that contaminated milk or byproducts imported from regions with high TB burden was used to produce these foods. *M. bovis* has been detected in milk, cheese and other products (Kaneene et al., 2014; Pereira-Suarez et al., 2014; Pérez-Lago et al., 2014; Cezar et al., 2016), mainly in regions where animal TB still represents a major issue.

All samples positive only to *Mycobacterium* sp. DNA corresponded to dairy products. This could be related to a higher exposure of milk to environmental NTM due to the way it is collected, transported and processed prior to being commercialized. The mycobacterial DNA detected displayed sequences highly compatible with *M*. *fortuitum* complex, *M*. *terrae* complex, and *M*. *gordonae*. These NTM are only occasionally considered pathogenic (Griffith et al., 2007). *M*. *fortuitum* complex is the only rapid growing mycobacterial group identified in our survey. However, it was detected in 4% of dairy samples, including one pasteurized milk with the highest estimated bacterial concentration (C_T_ = 32.64). The detection of these mycobacterial groups in unpasteurized milk is not unusual, but only few studies report on the isolation from pasteurized milk (Sgarioni et al., 2014), consistent with thermal inactivation profiles of *M*. *fortuitum* in milk (Grant et al., 1996).

PCR and further methods revealed *M*. *avium* subsp. *hominissuis* DNA in 2% of meat foodstuffs. Culture retrieved one *M*. *avium* subsp. *avium* isolate from a fresh chicken sausage, one *M*. *avium* subsp. *hominissuis* isolate from ground beef and one *M*. *senegalense* isolate from mortadella. These data diverged from other surveys on *M*. *avium* subspecies detection showing quite high proportions of PCR positives but no isolate recovery (Klanicova et al., 2011; Lorencova et al., 2014). The outcome of our market situation study may fit best with other reports (Jaravata et al., 2007; Savi et al., 2015). *M*. *avium* isolates were grown from uncooked meat products. *M*. *senegalense* strain was isolated from a cold cut originated from cooked pork meat and fat, suggesting an environmental contamination during further processing. *M*. *avium* subsp. *avium* is an obligate pathogen rarely isolated from environmental samples unrelated to birds (Turenne et al., 2008). The subspecies *avium* is the main cause of typical avian TB in domestic birds and can infect cattle, deer and wild boar, and humans more sporadically (Dvorska et al., 2004). Since these infections are more common in older animals, we find more likely that this sausage contained meat from culled laying hens instead of young broilers specifically bred for meat and deduce that improved control measures are desirable. Raw meats are generally intended for being consumed cooked, but eating undercooked meats is quite common and mycobacterial inactivation during cooking is time and temperature dependent (Hammer et al., 2013). In addition, cooked products can become cross-contaminated through direct or indirect contact with raw meat as a consequence of improper food handling. Meat could still pose a risk of exposure to viable *M*. *avium* subspecies and other mycobacteria if not produced hygienically and cooked properly (Klanicova-Zalewska and Slana, 2014).

Mycobacteria are present infecting animals and in our environment, water and food being likely means of contact for humans. Zoonotic TB and NTM infection have been of great importance in human health over time. The prevalence of zoonotic TB was dramatically reduced owing to the implementation of abattoir inspection, milk pasteurization and other measures, but the problem is still considerable in many regions (Kaneene et al., 2014). With respect to NTM, much effort has been devoted to the understanding of human infection and thus different mechanisms leading to susceptibility and predisposition consisting in immunological flaws and conditions that compromise physical barriers to infection have been identified (Lake et al., 2016). Exposure to attenuated or inactivated mycobacteria can modulate the immune response resulting in a favorable effect (Beltran-Beck et al., 2014; Cardona et al., 2015; Kaufmann et al., 2015; Chambers et al., 2017). On the other hand, genetic susceptibility to chronic inflammatory disorders coupled with heavy exposure to *M. avium* subsp. *paratuberculosis* has been pointed out as a potential etiological factor in Crohn's and other chronic inflammatory diseases (Sechi and Dow, 2015). Moreover, it has been hypothesized that it could drive an autoimmune response by molecular mimicry (Sechi and Dow, 2015). Unfortunately, a consensus opinion on the potential role of this bacterium in Crohn's disease has not yet been reached (Van Kruiningen, 2011; Waddell et al., 2016). Since few bacteria are detected in Crohn's disease patients it could be speculated that *M*. *avium* subsp. *paratuberculosis* might not need to be active to trigger a deleterious response. A latent, dormant or even dead condition could be sufficient as showed by the necrotizing colitis induced in mice after transanal injection of *M*. *avium* subsp. *paratuberculosis* antigens (Momotani et al., 2012). However, our results do not indicate that a heavy foodborne exposure occurs in populations where the main source of food of animal origin is processed food.

In summary, our results show that the prevalence of viable mycobacteria in packed products of animal origin available at Spanish supermarkets was not substantial although a considerable proportion of them contained mycobacterial DNA. These figures are probably translatable to other European countries as a result of market globalization and common food safety legislation. This survey indicates that viable mycobacteria and mycobacterial components are present in a range of products and at a frequency that could ensure repeated exposure over time during the lifetime of any individual. Consequently, setting up more stringent control measures should be considered. Further research is necessary to identify critical points in the food production system and determine the real impact of foodborne mycobacterial exposure on human health either directly, causing infection, or indirectly, modifying individual's immune status and susceptibility to other diseases.

## Author contributions

IS, RJ, and JG conceived of the study. EM, MT, IS, and NE carried out the laboratory work and compiled and analyzed the data. IS collated the results and wrote the original draft. IS, EM, MT, NE, RJ, and JG participated in the review and the editing of the original draft. All authors read and approved the final manuscript.

### Conflict of interest statement

The authors declare that the research was conducted in the absence of any commercial or financial relationships that could be construed as a potential conflict of interest.
